# A case report of Behcet's disease in a child with trisomy 8 and literature review

**DOI:** 10.3389/fped.2025.1750552

**Published:** 2026-01-30

**Authors:** Kaixi Zhang, Chenxi Wei, Lijun Jiang, Xue Zhao, Qingxiao Su, Xingjie Qi, Hui Zhao, Zanhua Rong

**Affiliations:** 1 Department of Pediatrics, Second Hospital of Hebei Medical University, Hebei Medical University, Shijiazhuang, China; 2 Key Laboratory of Rare Diseases of Hebei Province, Shijiazhuang, China

**Keywords:** autoinflammation, Behcet's disease, children, mosaicism, trisomy of chromosome 8

## Abstract

**Objective:**

Report a case of Behcet's disease (BD) in a child with trisomy 8 mosaicism (T8M), providing insights for the diagnosis, treatment, and prognosis of this condition.

**Methods:**

The clinical data of an 11-year-old girl with T8M mosaicism and BD who was admitted to our hospital in August 2022 were retrospectively analyzed, and the relevant literature was reviewed.

**Results:**

The main clinical manifestations of the child were recurrent oral ulcers, vulvar ulcers, fever, and joint deformities of both hands. Inflammatory markers (CRP, white blood cell, IL-6, IL-17) were significantly increased, and complement C3 and C4 were decreased. Cranial MRI showed dysplasia of the corpus callosum and ventriculomegaly. BD was diagnosed according to the international standard, and genetic testing confirmed a karyotype of 47, XX, +8[9]/46, XX [31] (chimerism ratio 22.50%). After treatment with glucocorticoids combined with immunosuppressants (including thalidomide, etc.), the ulcer and inflammatory markers were rapidly relieved. However, the ulcer recurred two and a half years after drug withdrawal.

**Conclusions:**

T8M may be detected in the autoinflammatory stage of BD, before any overt hematologic manifestations appear. Children presenting with BD-like features plus developmental anomalies or refractory autoinflammation should undergo karyotyping to establish the presence or absence of T8M, thereby informing both clinical management and genetic counseling.

## 1 Introduction

Mosaicism of trisomy 8 syndrome (T8M), also known as Warkany syndrome, is a rare chromosomal abnormality with an estimated incidence of between 1 in 25,000 and 1 in 50,000 live births and a significantly higher prevalence in males than in females (approximately 5:1) (1). Complete non-mosaic trisomy usually results in an early miscarriage. Therefore, most surviving cases are mosaic. The clinical manifestations of trisomy are highly heterogeneous and may involve multiple systems. Common features include distinctive facies (e.g., prominent forehead, hypertelorism, and low nasal bridge), mild to moderate intellectual disability and developmental delay, deep plantar and palm fold, skeletal abnormalities (e.g., joint contractures, vertebral malformations), congenital heart defects, renal abnormalities, and agenesis of the corpus callosum (2–4).

T8M can be divided into congenital and acquired types based on its mechanism of occurrence. Constitutional trisomy 8 mosaicism (cT8M) typically arises from postzygotic mitotic errors, such as chromosomal nondisjunction, or structural anomalies including pseudo dicentric chromosome 8. This results in widespread distribution of trisomic cell lines across various tissues, which represents a primary clinical etiology for multiple congenital malformations and intellectual disability (5). In contrast, the acquired type is a clonal chromosomal aberration that is acquired, which is mainly associated with hematopoietic malignancies, especially myelodysplastic syndrome (MDS) and acute myeloid leukemia (AML) (6). It is worth noting that acquired T8M is not only closely associated with hematological malignancies, but also accompanied by systemic autoinflammatory manifestations, such as periodic fever and Behcet's disease (7). The underlying mechanism may involve immune dysregulation. Trisomic clones, particularly within the myeloid lineage, may exhibit a constitutively active immune state. This can manifest as enhanced innate immune responses via signaling pathways such as those involving Toll-like receptors (8), overproduction of pro-inflammatory cytokines (e.g., IL-1β, IL-6, TNF-α), and potentially aberrant type I interferon signaling (9). Furthermore, the trisomic mosaic cells residing in immune organs or vascular tissues may themselves act as persistent endogenous danger signals. This presence can break immune tolerance and drive inflammatory responses against self-tissues, thereby linking localized chromosomal mosaicism to systemic autoinflammation (10).

**Figure 1:**
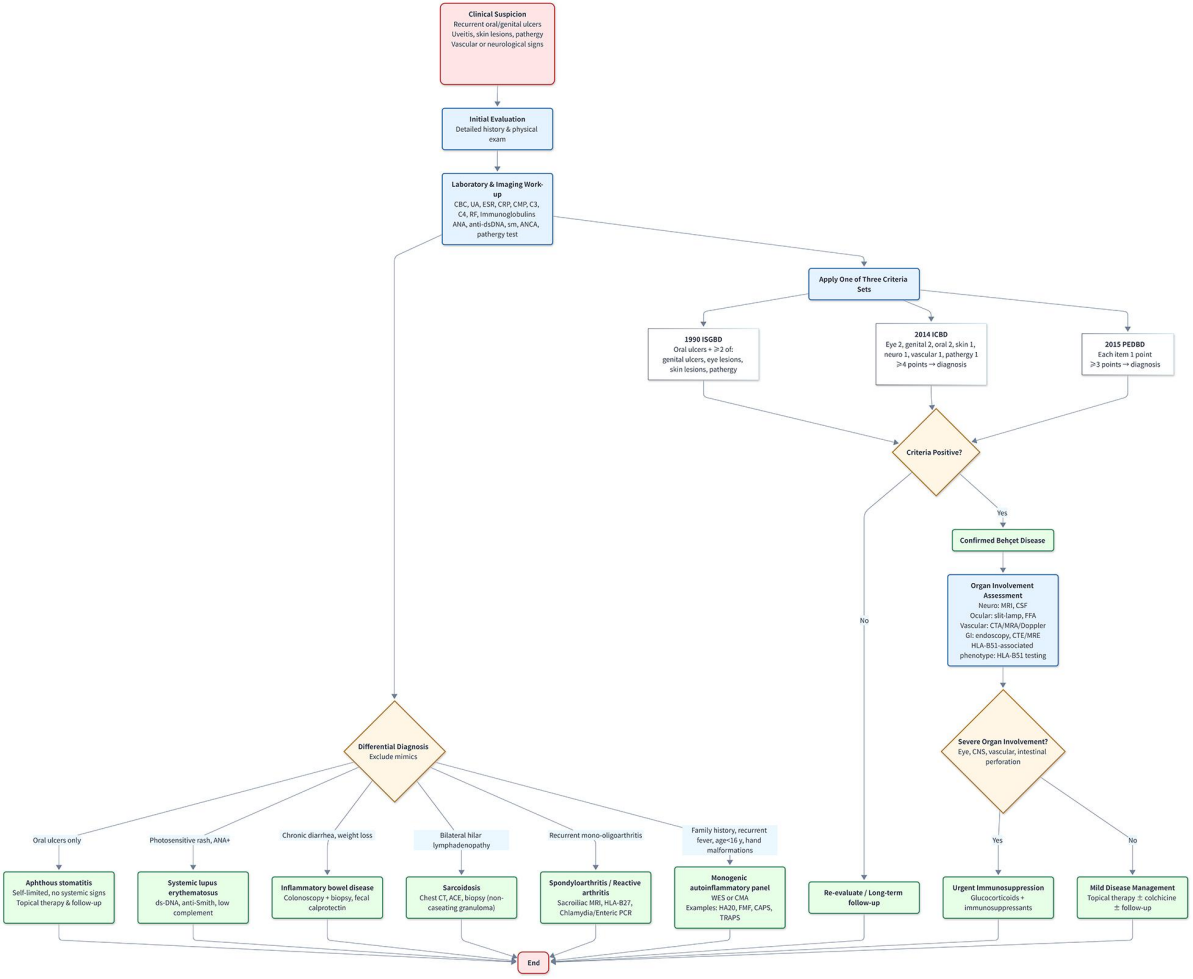
Diagnostic and differential algorithm for Behçet's disease. ISGBD (20), International study group for Behçet's disease (1990 criteria); ICBD (21), International criteria for Behçet's disease (2014 criteria); PEDBD (22), pediatric classification criteria for Behçet's disease (2015 criteria); CBC, complete blood count; UA, urinalysis; ESR, erythrocyte sedimentation rate; CRP, C-reactive protein; CMP, comprehensive metabolic panel; C3/C4, complement components 3 and 4; RF, rheumatoid factor; ANA, antinuclear antibody; anti-dsDNA, anti-double-stranded DNA; ANCA, anti-neutrophil cytoplasmic antibody; WES, whole-exome sequencing; CMA, chromosomal microarray; MRI, magnetic resonance imaging; CSF, cerebrospinal fluid; FFA, fundus fluorescein angiography; CTA, computed tomography angiography; MRA, magnetic resonance angiography; MRE, magnetic resonance enterography; HLA-B51, human leukocyte antigen-B51.

Behcet's disease is a chronic, recurrent systemic vasculitis that affects both arterial and venous systems (11). The typical clinical presentation includes recurrent oral ulcers, genital ulcers, skin lesions, and ocular inflammation (e.g., panuveitis with retinal vasculitis). The etiology of BD is not completely clear, and may be related to infection, genetic and immune factors (Figure 1). In this case, the child presented with isolated Behçet's disease symptoms without any evidence of blood system involvement, an unusual finding that highlights the significant heterogeneity in T8M-related clinical manifestations.

## 2 Case report

An 11-year-old girl was admitted with a 4-year history of recurrent oral ulcers, vulvar ulcers noted 2 weeks earlier, and fever for 1 day. Episodes of oral aphthosis began insidiously four years ago, occurring every 1–2 months as single, painful ulcers on the oral mucosa or tongue tip that healed spontaneously within 10 days without specific therapy. Two weeks before admission, painless vulvar ulcers were observed and left untreated. One day prior to admission she developed fever; at the local hospital she received intravenous piperacillin–tazobactam, dexamethasone, and local ulcer care. For further evaluation she was transferred to our unit with a provisional diagnosis of Behçet's disease. Since onset she has maintained normal appetite and sleep, reported no bloody diarrhea or arthralgia, and has had no growth or developmental delay. Antenatal, perinatal, feeding, past medical, personal, and family histories were unremarkable.

**Figure 2:**
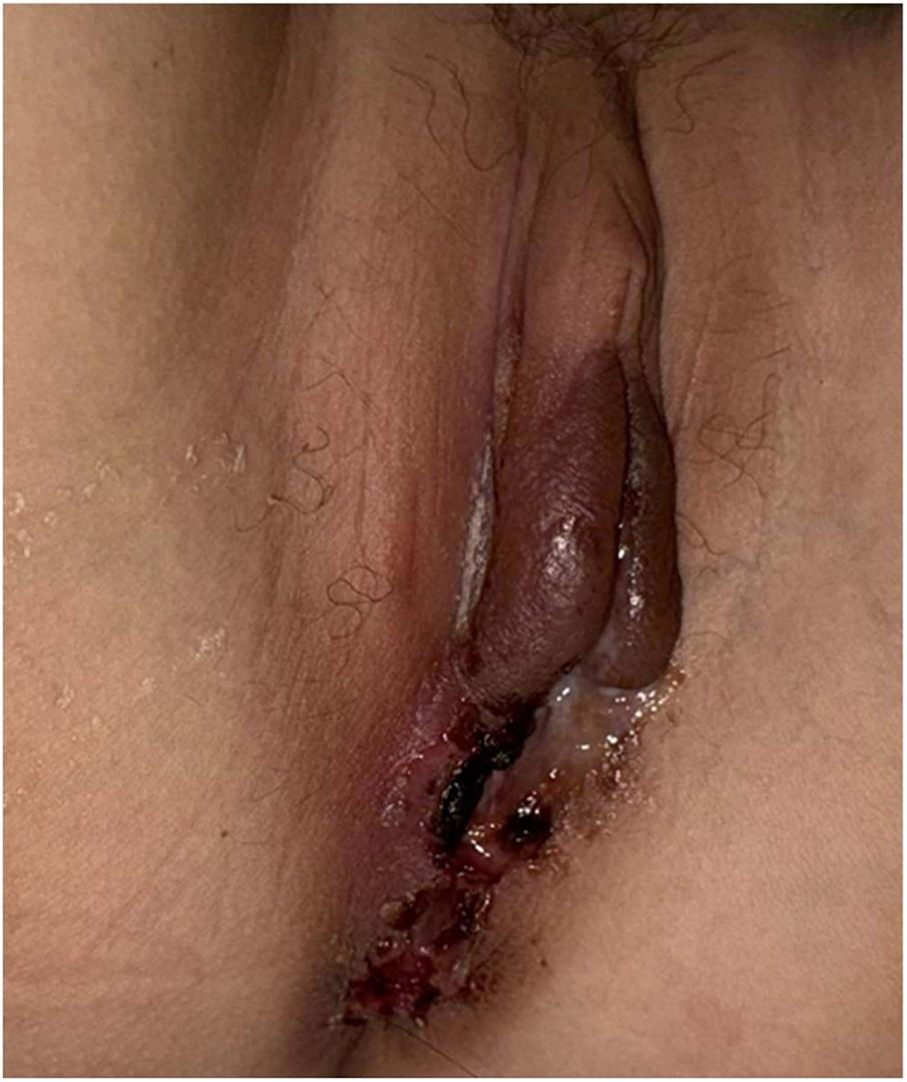
Clinical photograph reveals erythema and crusting of the left labium majus with scant mucopurulent exudate. Written informed consent for publication was obtained from the patient's legal guardian.

**Figure 3:**
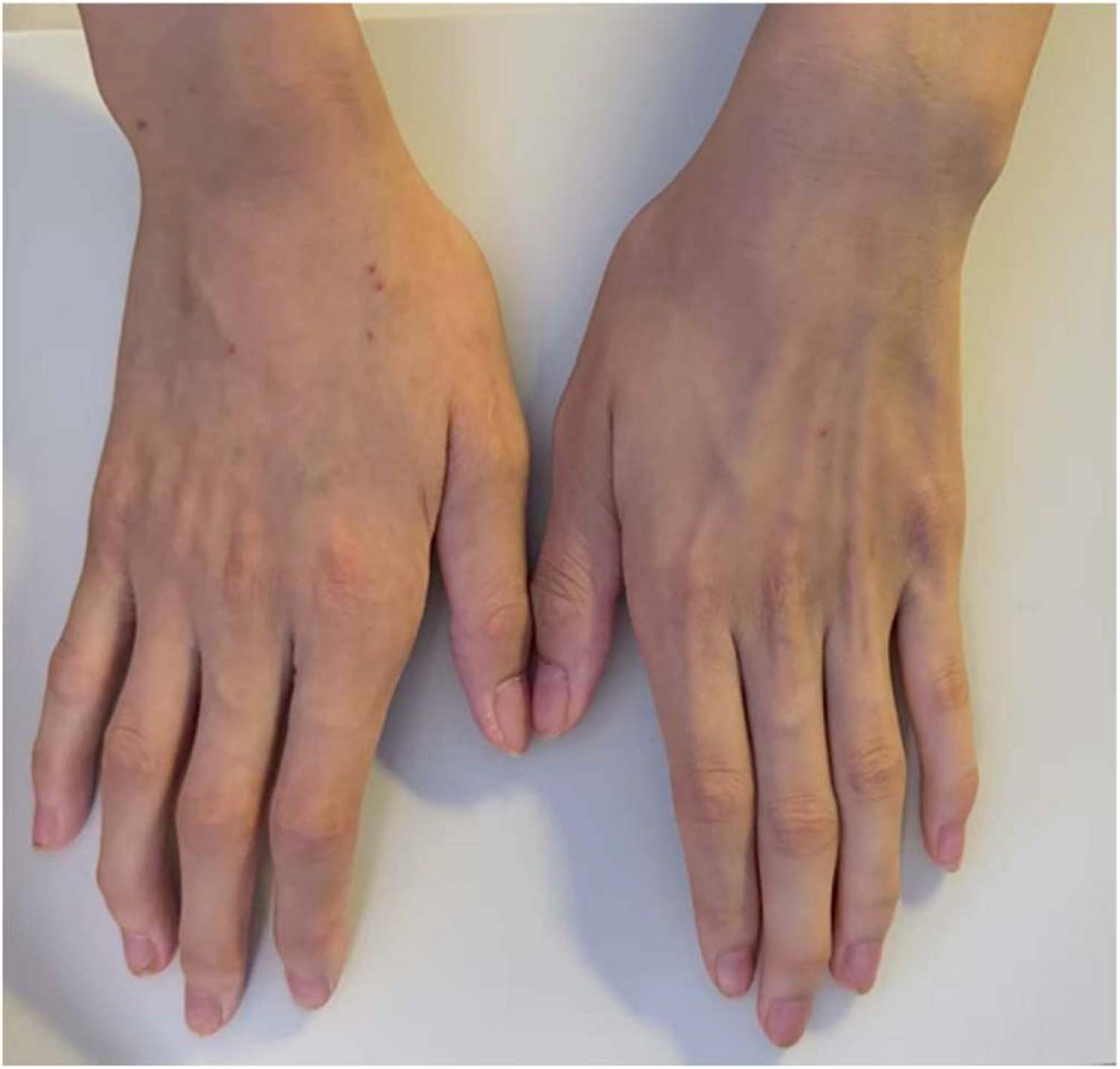
Fixed flexion deformities of the metacarpophalangeal and proximal interphalangeal joints in both hands. Written informed consent for publication was obtained from the patient's legal guardian.

Physical examination: height 154.00 cm, weight 37.00 kg. Discrete aphthous ulcers were scattered throughout the oral mucosa. The left labium majus showed erythema, induration, and crusting with a scant mucopurulent exudate (Figure 2). Flexion contractures of the metacarpophalangeal and proximal interphalangeal joints were noted bilaterally, with limited active extension (Figure 3).

Auxiliary investigations: Inflammatory markers showed elevated high-sensitivity C-reactive protein (65.90 mg/L), whereas erythrocyte sedimentation rate was within the normal range. Complete blood count revealed leukocytosis (16.82 × 10⁹/L) and neutrophilia (12.30 × 10⁹/L). Cytokine profiling demonstrated up-regulation of IL-2 (9.54 pg/mL), IL-4 (31.21 pg/mL), IL-5 (9.04 pg/mL), IL-6 (22.60 pg/mL), IL-10 (36.96 pg/mL), IL-12p70 (27.55 pg/mL), IL-17 (183.63 pg/mL) and IFN-α (14.87 pg/mL). Lymphocyte subsets disclosed an increased absolute CD19⁺ B-cell count (618.00/μL) and a reduced CD16⁺CD56⁺ NK-cell count (96.00/μL). Complement C3 (0.59 g/L) and C4 (0.11 g/L) were both below the reference limits. Urinalysis, stool routine, procalcitonin, ferritin, serum immunoglobulins, coagulation screen, comprehensive metabolic panel, virological tests (EBV, CMV), QuantiFERON-TB, autoantibodies, fecal calprotectin, rheumatoid triad (RF, ASO, anti-CCP) and HLA-B51 were all unremarkable (as shown in Table 1).

**Table 1:** Dynamic laboratory parameters in the present case across active and remission phases.

Laboratory index	Acute phase （2022-8-15）	Stable phase （2023-8-17）	Relapse phase （2025-4-26）	Stable phase （2025-9-18）
WBC (*10^9^/L)	16.82	7.20	9.99	8.37
NE# (*10^9^/L)	12.30	4.72	8.08	6.07
LY# (*10^9^/L)	2.67	1.68	1.06↓	1.71
NE# (%)	73.11	65.61	80.9↑	72.48
LY# (%)	18.54	23.35	10.59↓	20.39
HGB (g/L)	128.00	135.00	112.00↓	114.00
PLT (*10^9^/L)	162.00↓	294.00	314.00	435.00
ESR (mm/h)	8.00	2.00	7.00	2.00
CRP (mg/L)	65.90↑	3.30	5.83	8.61
C3 (g/L)	0.59↓	0.71	1.17	/
C4 (g/L)	0.11	0.12	0.31	/
IL-1β	11.76	<2.5	1.89	/
IL-2	9.54↑	/	1.74	/
IL-4	31.21↑	<2.5	2.94	/
IL-5	9.04↑	/	4.56	/
IL-6	22.60↑	3.36	1.18	/
IL-8	19.50	<2.5	2.05	/
IL-10	36.96↑	2.97	1.65	/
IL-12p70	27.55↑	/	/	/
IL-17	183.63↑	/	1.29	/
IFN-α	14.87↑	/	1.75	/
IFN-γ	12.42	/	2.83	/
TNF-α	14.87↑	3	1.89	/

WBC, white blood cells; NE#, absolute neutrophil count; LY#, absolute lymphocyte count; NE%, neutrophil percentage; LY%, lymphocyte percentage; HGB, hemoglobin; PLT, platelets; ESR, erythrocyte sedimentation rate; CRP, C-reactive protein; C3/C4, complement components 3 and 4; IL, interleukin; IFN, interferon; TNF-α, tumor necrosis factor-alpha.

Imaging and specialized tests: Pathergy test was positive. Esophagogastroduodenoscopy and colonoscopy revealed no abnormalities. Brain MRI suggested possible hypoplasia of the corpus callosum and cerebral parenchyma, accompanied by dilatation of the atria and temporal horns of both lateral ventricles; cerebral MRA was normal, whereas the left transverse and sigmoid sinuses were hypoplastic. Abdominal ultrasound showed mildly thickened and irregular gallbladder wall; liver, pancreas, spleen and kidneys were free of masses, several enlarged mesenteric lymph nodes were noted, and intestinal loops were normal. Echocardiography demonstrated mild mitral and tricuspid regurgitation. Lower-limb arterial ultrasound revealed decreased flow velocity in bilateral dorsalis pedis arteries; remaining upper- and lower-limb arterial and venous Doppler studies were normal. Chest CT showed minimal thickening of the right interlobar pleura; no other abnormalities were detected. Electrocardiography showed sinus rhythm with occasional atrial premature complexes, some conducted with aberrancy.

**Figure 4:**
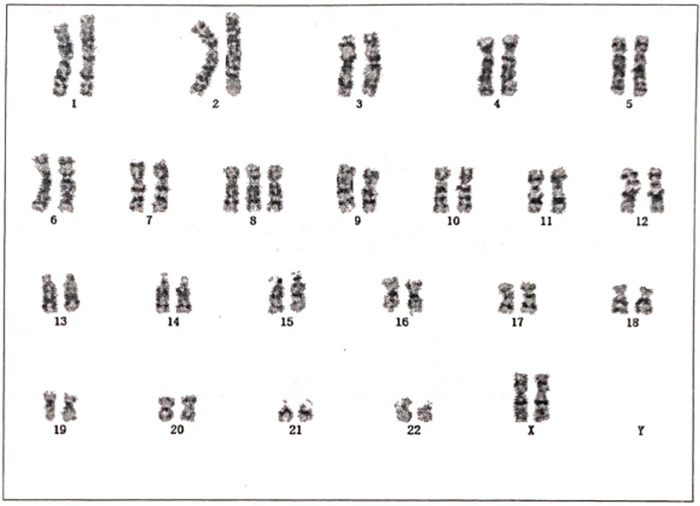
Karyotype report of peripheral blood G-banding analysis showing mosaic trisomy 8 with the karyotype 47, XX, +8([9])/46, XX ([31]).

Genetic analyses: Conventional karyotyping of peripheral-blood lymphocytes established the diagnosis of trisomy 8 mosaicism: 47, XX, +8([9])/46, XX ([31]) (Figure 4). Chromosomal microarray analysis (CMA) disclosed a 146.17 Mbp duplication encompassing 8p23.3-q24.3 [seq [GRCh37] dup (8) (p23.3q24.3)], spanning almost the entire chromosome 8 and providing molecular confirmation of mosaic trisomy 8.

Treatment and follow-up: On admission, the patient was started on oral prednisone 30 mg once daily, thalidomide 25 mg once daily, and hydroxychloroquine 0.1 g twice daily. One month later, oral and vulvar ulcers had markedly improved, with no purulent exudate; high-sensitivity C-reactive protein had normalized. Prednisone was tapered according to protocol and discontinued within two months. Thirty months after steroid withdrawal, vulvar ulcers recurred; the patient was readmitted and treated with intravenous methylprednisolone sodium succinate, thalidomide, and adalimumab for one week, resulting in complete remission. She remains under regular follow-up; to date, her disease is stable and no hematologic abnormalities have emerged.

## 3 Discussion and conclusions

This report describes a pediatric patient who presented with classic Behçet's disease (BD) manifestations and joint contractures and was ultimately diagnosed with trisomy 8 mosaicism (T8M). Notably, despite a substantial proportion of trisomic cells, the child has not developed MDS or any other hematologic abnormality—an outcome that contrasts sharply with the frequent clonal hematopoiesis observed in adults with acquired +8.

We systematically searched PubMed for reports of pediatric trisomy 8 (T8) co-existing with Behçet's disease (BD) using the keywords “Trisomy 8” and “Behçet's disease”, and retained patients aged <18 years without hematological involvement (12–15). Three additional Chinese-language cases reported by Zhao Wan-wen et al. that are not indexed in PubMed were included (16). In total, eight T8 children (one from the present study) fulfilled the inclusion criterion of no hematological disease (as shown in Table 2).

**Table 2:** Summary of pediatric cases of T8M with BD (present case included).

General condition	1	2	3	4	5	6	7	8
Gender	F	F	F	F	M	M	F	F
Age of onset	11	14	3	4	2	2	14	7
Age at diagnosis of BD	11	14	6	7	7	-	15	13
Age at diagnosis of T8	12	14	6	7	7	12	15	13
Malignant hematological diseases combined	N	N	N	N	N	N	N	N
Mucocutaneous	Oral ulcer, Genital ulcer	Oral ulcer, Genital ulcer	Oral ulcer, Genital ulcer	Oral ulcer, Ocular symptom, Erythema nodosum	Oral ulcer	Oral ulcer	Oral ulcer, Genital ulcer, Psoriasis	Oral ulcer, Genital ulcer, Scaly skin lesions
Gastrointestinal	N	–	Y	–	Y	Y	–	–
Neurologic	Joint deformities	Developmental delay, Intellectual disability, Joint deformities		Developmental delay, Intellectual disability, Deep palmar		Joint deformities	Joint deformities, Deep palmar	
Immunological	–	–	Fever	Fever, Arthritis/Joint pain	Fever	Arthritis/Joint pain		–
Accessory examination								
hypocytosis	Y	N	N	Y	N	N	N	N
Inflammatory index	CRP	CRP	CRP, ESR	ESR	ESR, FCAL	ESR, FCAL	CRP, ESR	CRP, ESR
Immune globulin	N	–	N	–	IgA↑, IgG↑, IgM↓	IgG↓, IgM↓	IgM↑	N
Alexin	C3↓,C4↓	–	–	–	–	–	C4↑	C3↑
Antinuclear antibodies	N	–	–	N	N	N	N	H, S
Treatment and prognosis								
Treatment	Pred, THD, HCQ	–	–	MP, Pred, CsA, AZA	5-ASA, SASP, Pred, THD	EEN,5-ASA, THD, AZA	TNFi	TNFi
Dead or not	N	N	N	N	N	N	N	N

CRP, C-reactive protein; ESR, erythrocyte sedimentation rate; FCA, flow-cytometric activated cell analysis; FL, ferritin; IgA/G/M, immunoglobulin A/G/M; C3/C4, complement components 3 and 4; HS, homogeneous pattern on ANA; Pred, prednisone; IHD, immunosuppressive high-dose therapy; HC, hydroxychloroquine; MP, methylprednisolone pulse; CsA, cyclosporine A; AZA, azathioprine; 5-ASA, 5-aminosalicylate; SAS, sulfasalazine; E, etanercept; TH, thalidomide; DA, dapsone; ZA, zoledronic acid; TNF, TNF-α inhibitor.

None of these patients exhibited anemia, cytopenia or other blood-system abnormalities, a profile that differs markedly from the frequent association of acquired +8 with MDS in adults. Females predominated (87.50%). Age at disease onset ranged from 2 to 14 years. Universal findings were oral aphthosis (100%); genital ulcers occurred in 62.50%, fever in 50.00%, whereas ocular inflammation was uncommon (12.50%). No patient developed a hematological malignancy; gastrointestinal and cutaneous lesions were documented in 37.50% and 50.00%, respectively.

Developmental anomalies included growth retardation, intellectual disability and joint contractures consistent with T8 mosaicism. Laboratory data showed cytopenia in 25.00% of cases, elevated inflammatory markers (CRP, ESR and/or fecal calprotectin) in the majority, and variable immunoglobulin and complement levels. Antinuclear antibodies were consistently negative except in one patient.

Therapeutic regimens comprised corticosteroids (methylprednisolone or prednisone), immunosuppressants (ciclosporin, azathioprine), 5-aminosalicylates (5-ASA, sulfasalazine), thalidomide and/or TNF-α inhibitors. All patients remain alive and clinically stable on follow-up.

These data delineate a distinct pediatric phenotype in which T8M with Behçet-like manifestations is driven by autoinflammation rather than hematologic neoplasia. The phenotypic distinctions potentially stem from differences in the chimeric distribution of the +8 clone *in vivo*. Cytogenetic studies indicate that trisomy cells are more readily detectable in non-hematopoietic tissues, such as oral mucosa, yet are nearly absent or present only in minute quantities in CD3⁺ T lymphocyte (17). This lineage-specific distribution disparity represents a fundamental biological distinction between cT8M and acquired +8.

Acquired +8 typically arises later in life as a somatic mutation, predominantly localized within the myeloid lineage (e.g., granulocytes and monocytes), and serves as a common clonal marker in MDS and AML (6). In contrast, cT8M constitutes a constitutional mosaic state formed during early postzygotic development. Resulting from mitotic errors in embryogenesis, the +8 clone is generated and stochastically integrated into multiple developing cell lineages. Such clones may settle in precursor cells fated to differentiate into skin and mucosal tissues, or may partly engraft in hematopoietic progenitor cells, only to be eliminated or selected against during the stringent developmental screening process of T lymphocytes (18).

These lineage-distribution data provide a conceptual framework for understanding the phenotypic gap between children and adults with T8 mosaicism. Neither our patient nor the seven pediatric cases identified in the literature exhibited hematological abnormalities, whereas adults with cT8M frequently harbor MDS. This discrepancy likely stems from the fact that, in pediatric patients, the +8 clone has not yet accumulated sufficient secondary genetic events within the hematopoietic system to drive malignant transformation, or because immune-deregulatory mechanisms in childhood more readily manifest as an isolated autoinflammatory phenotype (19). Nevertheless, the neoplastic potential of the cT8M clone persists. Consequently, lifelong hematological surveillance is mandatory for children with confirmed cT8M. Regular full blood counts and peripheral-blood morphological reviews should be performed to ensure early detection of clonal evolution or progression to MDS/AML, enabling timely intervention.

Given the phenotypic complexity and long-term risks associated with T8M, any patient who presents with Behçet-like features or developmental anomalies and in whom conventional cytogenetics detects a + 8 clone should undergo lineage-specific mosaicism mapping. Fluorescence *in situ* hybridization (FISH) on sorted CD3⁺ lymphocytes and buccal mucosal cells is the method of choice. If the +8 signal is absent in peripheral-blood T cells but demonstrable in mucosal or myeloid compartments, the diagnosis of cT8M is strongly supported—a distinction that critically informs prognosis, genetic counselling, and treatment strategy.

## Data Availability

The original contributions presented in the study are included in the article/Supplementary Material, further inquiries can be directed to the corresponding author.
